# Editorial: Plants and environmental threats

**DOI:** 10.3389/fbioe.2024.1320759

**Published:** 2024-04-08

**Authors:** Ahmed A. Abdelhafez, Mohamed H. H. Abbas, Li Zhou

**Affiliations:** ^1^ Soils and Water Department, Faculty of Agriculture, New Valley University, New Valley, Egypt; ^2^ National Committee of Soil Science, Academy of Scientific Research and Technology, Cairo, Egypt; ^3^ Soils and Water Department, Faculty of Agriculture, Benha University, Benha, Egypt; ^4^ Eco-Environmental Protection Research Institute, Shanghai Academy of Agricultural Sciences, Shanghai, China; ^5^ Shanghai Engineering Research Centre of Low-Carbon Agriculture, Shanghai, China

**Keywords:** plants, environment, agriculture, sustainability, challenges, threats and attacks

In the face of escalating environmental challenges that threaten the delicate balance of our planet, the Research Topic titled “Plants and Environmental Threats” emerges as a beacon of hope and a pivotal contribution to the global discourse on ecological sustainability. This Research Topic of manuscripts transcends the boundaries of conventional academic compilations, embodying a confluence of innovative research, forward-thinking strategies, and an unwavering commitment to environmental stewardship. Within its pages, these studies weave a rich tapestry of knowledge that illuminates the indispensable role of plants and microbes in mitigating environmental threats, charting a course towards a sustainable future that hinges on our understanding of and collaboration with the natural world.

**FIGURE 1:**
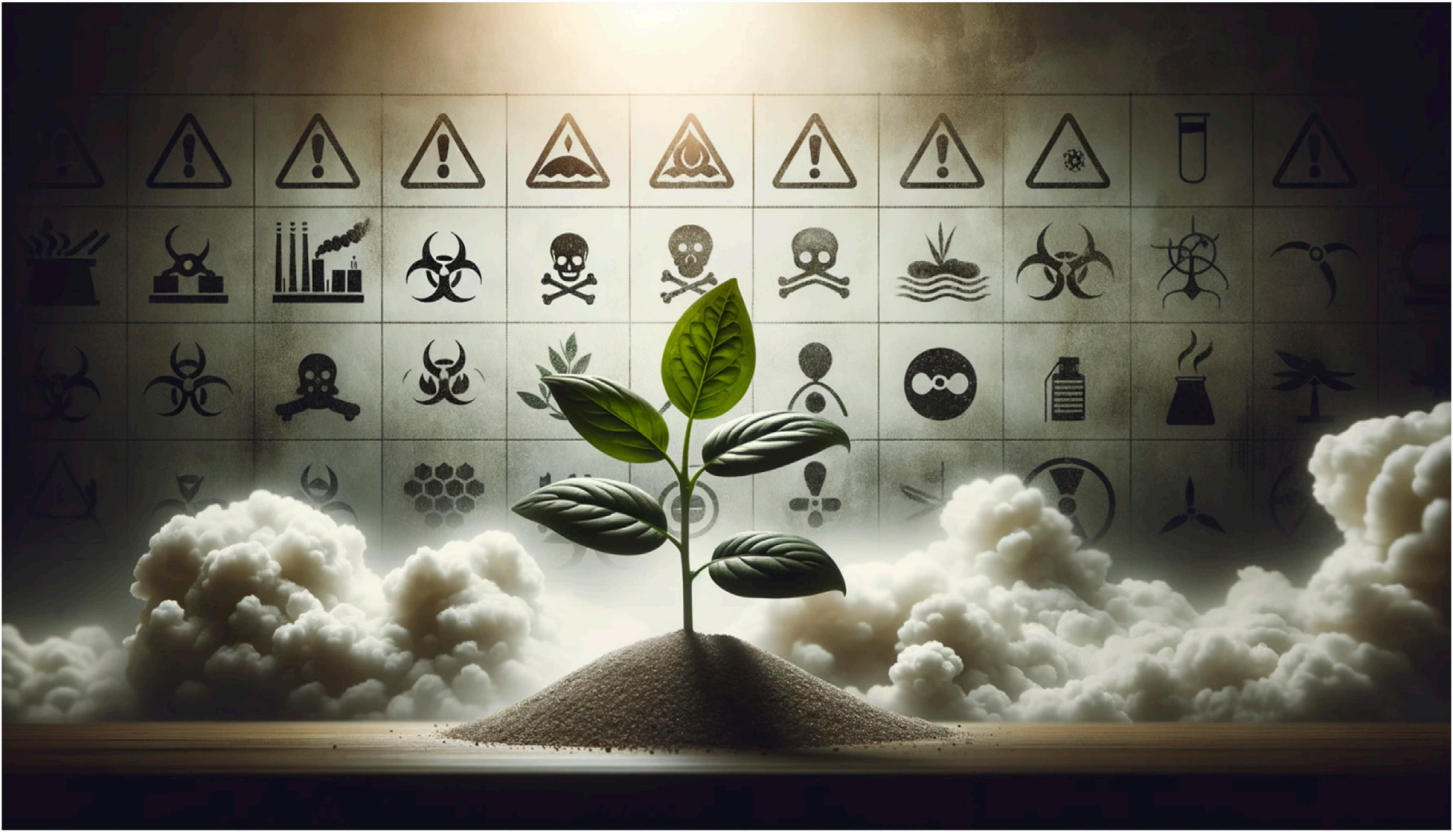
Plants and environmental threats.

As the foundation of most ecosystems on Earth, plants provide essential services including food production, carbon sequestration, and oxygen generation. However, escalating environmental perils pose growing risks to plant life across the globe. Recent scientific research illuminates both the emerging threats to plants as well as some promising biotechnology solutions. By examining key examples, we can understand the nuanced interplay between plants and their surrounding environments.

A study focusing on the yeast Kre6 in the marine alga *Pleurochrysis haptonemofera* provided new insights into the biochemical pathways involved in algal cell wall synthesis (Inukai et al.). The researchers found that the Kre6 homolog in P. *haptonemofera*, dubbed PhTGS, plays an essential role in assembling the β-glucans that provide structural integrity to algal cell walls. This study sheds light on the composition and formation of cell walls, which provide protection against environmental stressors. As climate change drives ocean warming and acidification, understanding algal cell wall biochemistry could enable cultivation of genetically improved strains with enhanced resilience to protect fragile marine ecosystems. Soil and water contamination from potentially toxic elements (PTEs) like lead, arsenic, and cadmium represents a massive environmental problem worldwide, but some innovative bioremediation techniques show promise in sustainably treating contamination. Furthermore, a review outlined how applying biochar, a substance produced by pyrolysis of organic waste biomass, can effectively immobilize PTEs in polluted soils (Zhang et al.). Biochar provides abundant adsorption sites via its porous structure and charged surfaces, which chemically bind free metal ions in soils. This reduces their mobility and bioavailability, thereby mitigating contamination of groundwater, uptake in food crops, and accumulation in the food chain. These properties make biochar a low-cost and eco-friendly solution to enable rehabilitation of soils in urban and mining areas that have been contaminated for decades. Ingenious applications of green nanotechnology are also harnessing the unique capabilities of plants and microbes for synthesis of nanomaterials. To what extent a bacterial species called *Bacillus paramycoides* could generate selenium nanoparticles with potent antioxidant and an antimicrobial effect was also highlighted in this Research Topic (Liu et al.). This green chemistry approach sustainably produces nanomaterials by turning simple selenium salts into nanoparticles using bacterial enzymes. The biogenic nanoparticles exhibited enhanced antibacterial activity against drug-resistant pathogens compared to conventional antibiotics. Such eco-friendly production of nanomaterials has diverse potential applications from medicine to environmental remediation. Beyond biosynthesis, the intrinsic biomolecules of organisms are inspiring advanced technologies as well. Recently in the journal Antibodies, researchers employed antibodies from alpacas to identify target proteins in the model organism *Drosophila melanogaster*, the common fruit fly (Qiu et al.). Camelid antibodies have enhanced heat-stability and solubility compared to traditional antibodies due to their unique nanobody structure. By bioengineering these robust nanobodies, researchers can create extremely sensitive and durable molecular probes for antigen detection in research and diagnostics.

Finally, rare earth metals have growing technological importance, but mining them causes substantial environmental damage. However, selected bacteria have a surprising appetite for rare earth elements (Paper et al.). These rare cyanobacteria can absorb and concentrate rare earths in their biomass, presenting opportunities to clean up mining wastewater contamination and even recover useful metals sustainably through biosorption.

In conclusion, while plants and ecosystems face escalating threats from pollution, climate change, and other anthropogenic impacts, biological research also reveals innovative solutions. With insightful science and responsible innovation, nature’s capabilities can be harnessed to create a cleaner, greener future. A nuanced understanding of the interconnectedness between plants and their environments will be crucial. The diverse perspectives and innovative approaches presented in these pages are vital in guiding our steps towards a more sustainable and ecologically balanced world. In closing, this Research Topic calls for creative thinking, responsible action, and collaborative efforts across disciplines to safeguard our planet for the wellbeing of future generations.

